# Notes and News

**Published:** 1900-12-01

**Authors:** 


### NOTES AND NEWS.

The Edinburgh and East Scotland Hospital has returned from South Africa.

Mrs. and Miss Avins have made a donation of 1,000 guineas to the Birmingham Orthopædic Hospital.

The members of the Children's Salon are endowing a cot at the North-Eastern Hospital for Children, to be named after the late Prince Christian Victor. An effort is being made to extend this most excellent hospital, for which £34,500 is needed.

A scheme is being formulated to provide a sanatorium for consumption at Winsley in Wiltshire for the combined counties of Wiltshire, Somerset, and Gloucestershire. The intention is that it shall be supported by voluntary subscription, and be built for the reception of 50 or 60 patients.

Mr. W. J. Crossley, of Manchester, is about to erect a sanatorium for consumption between Halkin and Rhydymwyn, in Flintshire, for the use of the county and district generally. The buildings will comprise a central residence surrounded by bungalows on a south-west slope to be used during the daytime. The air is dry and the country wooded with pine trees.

The Dumb Friends' League is making an appeal for the purpose of establishing ambulances for horses throughout the Metropolis. At present when a horse is disabled in the streets a considerable time must elapse before it can be removed, and then the means obtained is frequently unsuitable, and causes unnecessary suffering. The response to the appeal has as yet been very small, but wider publicity will doubtless secure enough at any rate to make a beginning.

Through a bequest of the late Mr. James Pyke Thompson, of Cardiff, his brothers, who are executors, have acquired Dulwich House, Canton, near Cardiff, and caused it to be converted into a convalescent home for women and children. The house is well adapted for the purpose, and is admirably situated, overlooking the public park maintained by Mr. Charles Thompson. It is to accommodate four women and eight children, patients being in part drawn from the Cardiff Infirmary. The executors are providing for the maintenance of the Home.

The Chelsea Hospital for Women has received a donation of £1,000 from H. M. E.

Dr. Thomas Buzzard will succeed Lord Lister as a representative of King's College upon the senate of the University of London.

The serious epidemic of typhoid which the Borough has been passing through is reported as having practically ceased. The character of the epidemic was mild, and no one course of contagion could be traced. The attack was worst in the quarter where the sanitation was most faulty.

Recent reports from Mauritius respecting the plague record upwards of 40 fresh cases of the plague. The percentage of deaths is very large. The type of case which has appeared at Williamstown, South Africa, appears to be mild and of a less rapidly contagious description than is usually the case.

Mrs. A. C. Bramble, of Nottingham, has bequeathed £1,000 to the Nottingham General Infirmary to endow a bed, £200 to the Eye Infirmary, £200 to the Dispensary, £200 to the Blind Institution of Nottingham, and £200 to the British Home for Incurables. Any residue of her estate is to go to the Nottingham General Hospital.

Last Saturday the Royal College of Surgeons in Ireland entertained Sir William Thomson and the Staff of the Irish Hospital in South Africa at Dublin. It was mentioned on the occasion that upwards of 5,000 cases had been treated in the hospital and at the dispensary, with a death-rate of only 3·7. Sir William Thomson stated that the Irish hospital was the only voluntary hospital sent out to Africa which was able to move of its own accord, having been equipped with the means of transport. The experience in consequence was very varied.

A case of interest to hospital managers came before the Queen's Bench on Monday. An action was brought before the Court for an injunction to restrain the collector of taxes from distraining on property belonging to St. Thomas's, St. Bartholomew's, and St. Bridewell Hospitals in respect to arrears of land tax. An Act was passed in the reign of George the Third whereby property belonging before 1693 to certain corporations, institutions, and charities were exempted from the payment of a land tax. Amongst the charities exempted are the institutions mentioned above. In virtue of the privilege so conferred the institutions resisted the demands made upon them, and in the result Mr. Justice Wills delivered judgment in favour of the hospitals.

One of the most important matters which came up at the meeting of the General Medical Council last Tuesday was the receipt of a communication from the Foreign Office in regard to the sale of false American diplomas, enclosing reports from Mr. W. Wyndham, the British Consul at Chicago. In a report dated July 3rd the Consul stated that the authorities had at last been able to arrest, and to hold to heavy bail for trial, Dr. Armstrong and his associates of the People's Institute, Van Buren Street, Chicago. The report proceeded to state that bogus diplomas were being sold all over Great Britain and British India, and probably in the Colonies. In a further report dated August 31st the Consul gave a list of persons "who appeared to have been duped by the diploma mill of Armstrong and Co., of Chicago." No doubt, he added, there were many others.

